# Contrast media for hysterosalpingography: systematic search and review providing new guidelines by the Contrast Media Safety Committee of the European Society of Urogenital Radiology

**DOI:** 10.1007/s00330-024-10707-6

**Published:** 2024-04-04

**Authors:** Remy W. F. Geenen, Aart J. van der Molen, Ilona A. Dekkers, Marie-France Bellin, Michele Bertolotto, Jean-Michel Correas, Gertraud Heinz-Peer, Andreas H. Mahnken, Carlo C. Quattrocchi, Alexander Radbruch, Peter Reimer, Giles Roditi, Carmen Sebastià, Fulvio Stacul, Laura Romanini, Olivier Clément, Torkel B Brismar

**Affiliations:** 1 Department of Radiology, Northwest Clinics, Alkmaar, The Netherlands; 2 Department of Radiology, Leiden University Medical Center, Leiden, The Netherlands; 3 AP-HP, University Hospital Bicêtre, Department of Radiology, BioMaps, University Paris Saclay, Le Kremlin-Bicêtre, France; 4 Department of Radiology, University Hospital Trieste, Trieste, Italy; 5 AP-HP, Groupe Hospitalier Necker, DMU Imagina, Service de Radiologie, Université de Paris, Paris, France; 6 Department of Radiology, Landesklinikum St Pölten, St Pölten, Austria; 7 Department of Diagnostic and Interventional Radiology, Marburg University Hospital, Marburg, Germany; 8 Centre for Medical Sciences - CISMed, University of Trento, Trento, Italy; 9 Clinic for Diagnostic and Interventional Neuroradiology, University Clinic Bonn, and German Center for Neurodegenerative Diseases, DZNE, Bonn, Germany; 10 Department of Radiology, Institute for Diagnostic and Interventional Radiology, Klinikum Karlsruhe, Karlsruhe, Germany; 11 Department of Radiology, Glasgow Royal Infirmary, Glasgow, UK; 12 Department of Radiology, Hospital Clinic de Barcelona, Barcelona, Spain; 13 Department of Radiology, Ospedale Maggiore, Trieste, Italy; 14 Department of Radiology, ASST Cremona, Cremona, Italy; 15 AP-HP, Hôpital Européen Georges Pompidou, DMU Imagina, Service de Radiologie, Université de Paris, Paris, France; 16 Unit of Radiology, CLINTEC, Karolinska Institutet, Alfred Nobels alle 8, 141 52 Huddinge, Sweden; 17 Department of Radiology, Karolinska University Hospital in Huddinge, Stockholm, Sweden

**Keywords:** Hysterosalpingography, Contrast media, Ethiodized oil, Adverse effects, Practice guideline

## Abstract

**Objectives:**

Hysterosalpingography (HSG) is widely used for evaluating the fallopian tubes; however, controversies regarding the use of water- or oil-based iodine-based contrast media (CM) remain. The aim of this work was (1) to discuss reported pregnancy rates related to the CM type used, (2) to validate the used CM in published literature, (3) to discuss possible complications and side effects of CM in HSG, and (4) to develop guidelines on the use of oil-based CM in HSG.

**Methods:**

A systematic literature search was conducted for original RCT studies or review/meta-analyses on using water-based and oil-based CM in HSG with fertility outcomes and complications. Nine randomized controlled trials (RCTs) and 10 reviews/meta-analyses were analyzed. Grading of the literature was performed based on the Oxford Centre for Evidence-Based Medicine (OCEBM) 2011 classification.

**Results:**

An approximately 10% higher pregnancy rate is reported for oil-based CM. Side effects are rare, but oil-based CM have potentially more side effects on the maternal thyroid function and the peritoneum.

**Conclusions:**

1. HSG with oil-based CM gives approximately 10% higher pregnancy rates.

2. External validity is limited, as in five of nine RCTs, the CM used is no longer on the market.

3. Oil-based CM have potentially more side effects on the maternal thyroid function and on the peritoneum.

4. Guideline: Maternal thyroid function should be tested before HSG with oil-based CM and monitored for 6 months after.

**Clinical relevance statement:**

Oil-based CM is associated with an approximately 10% higher chance of pregnancy compared to water-based CM after HSG. Although side effects are rare, higher iodine concentration and slower clearance of oil-based CM may induce maternal thyroid function disturbance and peritoneal inflammation and granuloma formation.

**Key Points:**

• *It is unknown which type of contrast medium, oil-based or water-based, is the optimal for HSG.*

• *Oil-based contrast media give a 10% higher chance of pregnancy after HSG, compared to water-based contrast media.*

• *From the safety perspective, oil-based CM can cause thyroid dysfunction and an intra-abdominal inflammatory response in the patient.*

**Supplementary Information:**

The online version contains supplementary material available at 10.1007/s00330-024-10707-6.

## Introduction

**Fig. 1:**
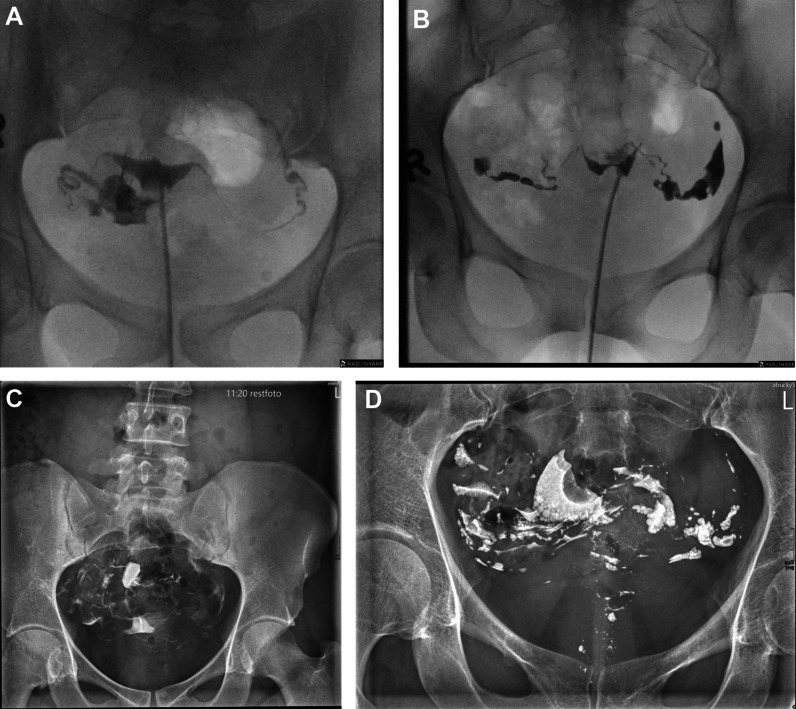
**A** Normal HSG examinations with symmetric filling of the fallopian tubes and peritoneal spill on both sides: **A** performed with iodixanol 320, and (**B**) performed with Lipiodol. Pelvic radiographs obtained 15 min post injection. Note the absence of droplet formation with iodixanol (**C**) and the presence with  Lipiodol (**D**). HSG technique: After cleaning of the perineum, a speculum is inserted into the vagina and the cervix is localized and cleaned. A catheter is positioned inside the cervical canal and an occluding balloon is inflated to the extent the patient can tolerate. After a scout radiograph of the pelvis with the catheter in place, contrast material is installed under fluoroscopic guidance and several spot radiographs are taken

Hysterosalpinography (HSG) is the radiological evaluation of the uterine cavity and the fallopian tubes (Fig. 1). The main indication is evaluation of infertility. Other indications include evaluation of tubal pathology, such as hydrosalpinx, salpingitis isthmica nodosa, and congenital anomalies and anatomic variants. Furthermore, recurrent spontaneous abortion, evaluation of tubal surgery, and preoperative evaluation prior to myomectomy are less common indications.

The iodine-based contrast medium (CM) used at HSG can be either oil-based or water-based. The only available oil-based iodine-based contrast medium today is Lipiodol Ultra Fluid (UF), which is composed of ethyl esters of iodine-based fatty acids of poppy seed oil [1, 2]. It was the world’s first iodine-based contrast medium, developed in 1901 by Marcel Guerbet [3]. In the 1960s, the original Lipiodol was chemically modified to Lipiodol UF, improving its fluidity and making it suitable for lymphography and other indications [4]. Lipiodol UF is available in 10-mL vials with an iodine concentration of 480 mg I/mL. The oil is not miscible in water and contains no additives [2]. In 2011, another oil-based CM used for HSG, Ethiodol, was taken out of production by its manufacturer [1]. Regarding water-based iodine-based CM, non-ionic CM have replaced the ionic CM in Western countries [5].

Although HSG is a widely used examination, controversies regarding the use of water or oil-based CM, and the associated fertility outcomes, remain today [6–9].

Therefore, the European Society of Urogenital Radiology (ESUR) Contrast Media Safety Committee (CMSC) undertook a systematic review on the evidence and external validity of reported pregnancy rates and CM-related complications and side effects to provide an up-to-date guideline.

## Methods

A systematic search in Medline was performed for studies reporting on HSG and CM. Two different search strategies were used: (A) For clinical trials and randomized controlled trials, literature was searched from 1980 to 2022; and (B) for (systematic) reviews and meta-analyses, the search was limited to the last 10 years.

Based on title and abstract, one reviewer (RG) with 24 years of experience in abdominal radiology selected the articles. Articles were selected when they described fluoroscopic HSG with a direct comparison for pregnancy rates and/or safety between oil- and water-based iodine-based contrast media. In total, this strategy resulted in nine clinical trials and ten reviews (Table 1). Cross-referencing was used when appropriate. Grading of the literature was performed based on the Oxford Centre for Evidence-Based Medicine (OCEBM) 2011 classification [10]. Special attention was paid to find randomized articles on HSG with two arms, one arm with oil-based CM and the other arm with water-based CM. The concept guideline was discussed by the CMSC members and consultants and approved at the CMSC Meeting in September 2023 in Rome (Italy).

**Table 1:** The literature search strategy

Keyword		Keyword	Trials	Reviews	Total included (trials + reviews)
Contrast media	**+**	HSG	51	17	8 + 5
		Tubal flushing	6	6	0 + 2
Complications	**+**	HSG	48	41	1 + 2
		Tubal flushing	3	1	0 + 0
		Lipiodol	88	28	0 + 0
Total					**9 + 10**

The number indicated in the last column describes the additional number of publications, not found by previous search terms. Eight randomized trials had a prime focus on pregnancy rates [11–18] and one on image quality and adverse effects [19]

## Results

Of the nine randomized found clinical trials, eight were mainly focused on pregnancy rates, and the last one had a focus on image quality [11–19]. Both the Cochrane review and the recent meta-analyses describe the quality of the included trials and possible biases in detail [7–9, 20], but do not focus on the specific type of CM used. Table 2 shows a summary focusing on the used CM in all published studies on HSG/tubal flushing comparing oil- versus water-based CM. Of the eight published studies, five used at least in one treatment arm a CM that is no longer on the market [11, 13, 15–17]. Only three studies used CM in both arms that are still commercially available [12, 14, 18]. Two studies compared Lipiodol UF to either ionic, high-osmolar diatrizoate [12] or diatrizoate, ioxaglate (ionic low-osmolar), and iohexol (non-ionic low-osmolar) [14].

**Table 2:** All published RCTs on HSG/tubal flushing with oil-based compared to water-based CM

Author	Year	Oil-based CM	Water-based CM	Pregnancies oil	Pregnancies water	*p* value
Schwabe et al [21]	1983	Ethiodol*	Sinografin*	11/56 (20%)	7/65 (11%)	NS
Alper et al [16]	1986	Lipiodol	Diatrizoate	14/46 (30%)	15/60 (25%)	NS
Boer de et al [22]	1988	Ethiodol*	Iopamidol	30/87 (34%)	23/88 (26%)	< 0.1
Rasmussen et al [17]	1991	Lipiodol	Diatrizoate Ioxaglate Iohexol	33%	Dia: 12% Iox: 17.6% Ioh: 20.8%	= 0.04
Lindequist et al [18]	1994	Lipiodol	Iotrolan*	29/121 (24%)	24/121 (20%)	= 0.44
Spring et al [23]	2000	Ethiodol*	Sinografin*	53/273 (19%)	54/260 (20%)	= 0.64
Dreyer et al [11]	2017	Lipiodol	Telebrix hystero*	220/554 (40%)	161/554 (29%)	< 0.001
Zhang et al [14]	2022	Lipiodol	Iohexol Iopromide Ioverol*	136/473 (29%)	96/479 (20%)	= 0.001

*Sinografin*: A mixure of 52.7% diatrizoate meglumine and 26.8% iodipamide meglumine. Both high osmolar ionic CM

*Telebrix hystero*: Ioxothalamic acid meglumine, a high osmolar ionic CM

*Diatrizoate*: High-osmolar, ionic CM. Branded under several names

*Iopamidol*: Low-osmolar, non-ionic CM. Branded under several names

*Ioxaglate*: Low-osmolar, ionic CM. Branded as Hexabrix

*Iohexol*: Low-osmolar, non-ionic CM. Branded as Omnipaque

*Iotrolan*: Iso-osmolar, non-ionic CM. Branded as Isovis

*Iopromide*: Low-osmolar, non-ionic CM. Branded as Ultravist

*Ioverol*: Low-osmolar, non-ionic CM. Only available in China

^*^Not commercially available

## Discussion

### Pregnancy rates

In 2020, a Cochrane review concluded that the available data on tubal flushing with oil-based CM versus water-based CM was too heterogeneous to perform a meta-analysis. Based on six randomized controlled trials (RCTs) with 2598 women included, authors concluded that oil-based CM may increase the odds of clinical pregnancy (OR 1.42, CI 1.10 to 1.85). This suggests that if the chance of pregnancy following tubal flushing with water-based CM is assumed to be 26%, the chance of pregnancy using oil-based CM would be between 28 and 39%. The quality of evidence was regarded as low [7].

The largest study included in this Cochrane review included 1108 women, of whom 554 women received 5–10 mL of Lipiodol, and the other 554 received 5–10 mL of Telebrix Hystero (Guerbet, France) [17]. This solution contains ioxithalamate meglumine, an ionic, high-osmolar CM with an iodine concentration of 250 mg/mL [24]. Within the European Union, Telebrix Hystero is currently only available in Belgium and Portugal [24]. Ongoing pregnancy, defined as a positive fetal heartbeat on ultrasound after 12 weeks of gestation, with the first day of the last menstrual cycle for the pregnancy within 6 months after HSG, occurred in 39.7% in the Lipiodol group and in 29.1% in the Telebrix Hystero group (Table 2, *p *< 0.001) [17]. At 5-year follow-up, a higher rate of ongoing pregnancy was observed with oil-based CM compared to water-based CM (80.0% vs. 75.0%), a higher rate of live births (74.8% vs. 67.3%), and a higher rate of naturally conceived pregnancies (41.4% vs. 34.7%) [25].

Since the Cochrane review, a large Chinese multicenter RCT by Zhang et al randomized a total of 1026 women to either Lipiodol (*n *= 508) or water-based CM (*n *= 518) [18]. The women in the water-based group received one of three low-osmolar iodine-based monomers with an iodine concentration between 300 and 320 mg I/mL (iohexol, iopromide, ioversol). Ongoing pregnancy within 6 months occurred in 29.1% after Lipiodol and in 20.1% after water-based CM (Table 2, *p *= 0.001). Live birth after > 24 weeks of gestation occurred in 169/473 women (36.1%) after Lipiodol versus 132/479 women (27.7%) after water-based CM (*p *= 0.006) [18]. This is comparable to the results of the aforementioned Dutch trial—the rate of pregnancies and live births after Lipiodol UF is approximately 10% higher than after water-based CM.

In Alper’s study, 15 of the 60 patients (25%) became pregnant after diatrizoate versus 14 of 46 patients (30%) after Lipiodol (non-significant difference) [12]. Rasmussen’s study had four arms with around 100 patients in each arm. The overall pregnancy rates were 12% for diatrizoate, 17.6% for ioxaglate, 20.8% for iohexol, and 33% for Lipiodol UF. Compared to the three water-based CM, Lipiodol had a significantly higher pregnancy rate (*p *= 0.04) [14].

Theoretically, non-ionic iso-osmolar CM seem to be the safest CM for HSG, as they exert no osmolar force. Only one study has researched the effect of the iso-osmolar iotrolan (withdrawn) versus Lipiodol [15]. No statistical difference in pregnancy rate was reported—29 of the 121 patients in the Lipiodol group became pregnant, compared to 24 of 121 patients in the iotrolan group [15]. Recently, the first RCT on HSG using the only commercially available iso-osmolar CM, iodixanol, has been announced [26].

One large RCT used an ionic high-osmolar CM as the comparing agent (Telebrix Hystero, withdrawn in 2018) [17, 24]. A review later raised concern that the comparative high-osmolar CM belonged to a superseded class of ionic CM with concentration, osmolality, and viscosity all lying at the extreme end [27].

### Image quality

On five image quality items (uterus opacification and outline, fallopian tube outline, visualization of fimbrial rugae, fallopian tube spillage, and peritoneal distribution), oil-based CM showed a significantly higher image quality than water-based CM [18]. A smaller study of 228 patients also showed a statistically overall better image quality score in the oil-based group versus water-based group [19]. In contrast, two smaller studies from the early 1990s showed significantly better image quality with water-based CM compared to oil-based CM [15, 28].

### Safety issues

The most common adverse effects of HSG, bleeding and infection, are independent on the type of CM used [6]. CM-related safety issues in HSG include hypersensitivity reactions, venous and lymphatic intravasation, effects on the peritoneal cavity, effects on the thyroid function of the women and conceived children, and infrequent complications published in case reports.

#### Hypersensitivity reactions

Hypersensitivity reactions during or directly after HSG are extremely rare. Exposure to CM can occur inside the female genital tract, due to venous and lymphatic extravasation and due to peritoneal spillage [29]. A national survey in the Netherlands reported on anaphylactic reactions and found no statistically significant difference in allergic reactions to HSG using oil-based and water-based CM (0.03% vs. 0.1%) [29]. Zhang et al also reported no observed hypersensitivity reactions [18].

#### Venous and lymphatic intravasation

Intravasation implies passage of injected CM from the uterine cavity to either myometrial vessels with subsequent drainage to pelvic veins (venous intravasation) or from the uterine cavity into draining myometrial lymph vessels with subsequent drainage to the lymph vessels of the broad ligament [30, 31]. Predisposing factors are tubal disease, recent uterine operation, uterine malformation, malplacement of the catheter, and excessive injection pressure or quantity of CM [32]. Women who experience pain during the procedure are more likely to show intravasation [31]. After venous intravasation, CM passes to the lungs where water-based CM quickly dissipates; no embolic symptoms or other side effects have been reported. Although concerns have been raised on the development of oil emboli after venous intravasation with oil-based CM, the general consensus is that oil emboli are innocuous and should not be considered a major complication [32]. Occasional symptoms such as chest pain, cough, dyspnea, light-headedness, confusion, headache, and very rarely cardiorespiratory failure and death have been described [32].

Intravasation has been reported between 0.4 and 6.9% [31]. Intravasation is the most commonly reported HSG complication, occurring in 2.7% of the patients imaged with oil-based CM and in 2.0% of the patients imaged with water-based CM [33]. Zhang et al reported a non-significant difference in intravasation between oil-based CM and water-based CM of 2.1 and 1.4% respectively [18]. Only the Dutch survey has reported a statistically significant difference in the incidence of intravasation between oil-based CM (4.8%) and water-based CM (1.3%, *p *< 0.001) [30].

However, there are four alarming reports that describe possible embolic complications of Lipiodol venous intravasation leading to cerebral, lung, and retinal emboli [34–37]. Onset of symptoms occurred between immediately after HSG to 6 days. In all four patients, emboli were suspected due to clinical status and physical examination but never proven. In one patient, who also had an episode of unexplained pancreatitis, pleural effusion showed high iodine concentration [36]. All four patients recovered after supportive treatment [34–37].

#### Effects on the peritoneal cavity

In contrast to water-based CM, oil-based CM are slowly absorbed by the fallopian tubes and the peritoneum. Reports from the 1930s and 1940s indicate that Lipiodol can persist in the female pelvis up to 22 months after HSG. In some of these patients, a foreign body reaction surrounding the Lipiodol rests has been reported [32]. The true incidence of granuloma formation around Lipiodol remnants in the female pelvis, and the clinical consequences are unknown [33].

#### Effects on the thyroid gland

Three Japanese studies addressed the effects of CM on the thyroid of the potential mother [38–40]. One of these is a comparative study on previously euthyroid women, where 164 patients underwent HSG with an oil-based CM and 94 with a water-based CM [36]. Subclinical hypothyroidism occurred in 22.6% of the women after oil-based CM and 9.5% after water-based CM (*p *< 0.05) [38]. Two other publications, where all women received Lipiodol, show similar results [39, 40]. Mekaru et al report that in 180 euthyroid women, 28 women developed subclinical hypothyroidism, two developed subclinical hyperthyroidism, and four developed asymptomatic hypothyroidism. All recovered without medication within 33 months. Ten of 28 women with subclinical hypothyroidism developed to hypothyroidism, while in the six women with subclinical hyperthyroidism, no significant changes occurred [39]. Kaneshige et al observed a significant increase in serum iodine concentration, urinary iodine/serum creatinine, and TSH in 22 previously euthyroid women at 4, 8, 12, and 24 weeks after HSG. These levels normalized after 9–12 months. Free triiodothyronine (FT_3_) and free thyroxine (FT_4_) levels were unaffected [40]. A study from New Zealand included 188 euthyroid women (95.9%) and eight with subclinical hypothyroidism (4.1%) [41]. After HSG, a urinary iodine concentration > 300 μg/L occurred in 98% of the women, peaking at weeks 1 and 4. After 24 weeks, urinary iodine concentration was still threefold higher than the recommended normal range upper limit and 121 women (61.7%) still had a concentration over the upper limit. TSH levels remained elevated up to 12 weeks, with a majority peaking in week 4. TSH levels rose above the upper normal limit in 71 women (37.8%), indicating subclinical hypothyroidism; FT_4_ levels stayed within the normal limits. Overt hyperthyroidism occurred in five women, and subclinical hyperthyroidism occurred in four women, developing later in the study period—between weeks 16 and 24 [41].

A systematic review on neonatal thyroid function after exposure to iodine-based CM by the mother prior to or during pregnancy estimated that the overall proportion of neonatal thyroid dysfunction after HSG was 0.0% [42]. In a follow-up study from the Dutch RCT, based on 138 consenting women, 76 neonates were conceived after HSG with Lipiodol and 64 with water-based CM [43]. No significant difference in T4 concentration was observed and none of the neonates had a positive screening result for congenital hypothyroidism [43]. A study from Japan reported that five of 212 infants (2.4%) conceived after HSG tested positive during congenital hyperthyroidism screening, compared to 0.7% in the entire screening group [44]. The mothers of these five infants received a significantly higher amount of Lipiodol during HSG, 20 mL versus 8 mL. Authors conclude that the dose of Lipiodol during HSG should be as low as possible to minimize the risk of neonatal thyroid dysfunction [44]. Finally, a study from New Zealand describes 146 neonates conceived within 6 months after Lipiodol HSG [45]. All babies had TSH levels within the normal range. Babies conceived during the first three cycles after HSG had the same range of TSH levels as those conceived in later cycles [45].

These results show that the risk of neonatal hypothyroidism after HSG is extremely low. Furthermore, many countries have screening programs for congenital hypothyroidism, so additional testing of neonates conceived after HSG is not indicated in those countries.

#### Infrequent complications

Two case reports from Japan describe two cases of fetal goiter after Lipiodol HSG [46, 47]. One resolved spontaneously before birth, the other within 4 weeks after birth. The relationship to Lipiodol exposure is unclear as average Japanese dietary intake of iodine is the highest in the world. It is postulated that Lipiodol can cause symptoms in women who have a high daily dietary iodine intake, due to the consumption of seaweed [47]. Iodine-induced hyperthyroidism after injection of a large amount (estimated 100 mL) of iodine-based CM under high pressure during HSG has been described. The type and brand of CM are not mentioned [48].

Two other reports address residual contrast material in the pelvis after HSG. In one case, the HSG contrast medium was not mentioned; in the other case, it was the oil-based Ethiodol. Both lesions were found accidentally, and both were operated, 6 months and 2 years after HSG. In the Ethiodol case, pathological diagnosis was a lipogranuloma. In the other case, no histopathology was performed, and the surgeon stated that it was elastic fatty tissue [21, 49].

The final report describes acute right upper quadrant abdominal pain, 2 days after HSG with non-specified oil-based CM [22]. Non-enhanced abdominal CT showed high-density oil on the surface of liver and spleen and in the uterine and pelvic cavity as well as small amounts of intraperitoneal free air. Authors called it a Fitz-Hugh-Curtis-like syndrome. The patient recovered after medical treatment [22].

## Future directions

Theoretically, water-based iso-osmolar CM are the safest CM for HSG, as they have no osmolar effect on the cells during flushing. The ideal RCT would be a comparison of HSG with Lipiodol UF and iodixanol 320 mg I/mL in infertile women with a low probability of tubal disease. A third study arm could include hysterosalpingo-contrast sonography in order to have a control group without iodine-based CM. Such an RCT should take pregnancies and live births into account, combined with maternal thyroid function before and after HSG, results of screening for hypothyroidism in the neonate, long-term follow-up of maternal thyroid function, and evaluation of abdominal complaints (chronic inflammation and abdominal adhesions).

## Conclusions

From the perspective of CM, the main problem is the limited external validity of the published studies. In five of eight studies, at least one treatment arm received a CM that is no longer on the market, so the published results are outdated, and cannot be controlled or repeated [11, 13, 15–17]. This means that of the five RCTs with Lipiodol UF, only three used CM that are still available in both arms (Table 1) [12, 14, 18]. Nevertheless, the largest and most recently published RCTs with 2134 included patients showed an increased pregnancy rate of 10% in the Lipiodol UF group compared to the water-based CM group [17, 18].

Caution is warranted when using Lipiodol in HSG. Lipiodol remains in the uterus, fallopian tubes, and peritoneal cavity for a prolonged period of time and can cause inflammatory changes in these tissues, although the incidence of these inflammatory changes and clinical consequences are unknown [32]. More evidence also exists regarding the effect of Lipiodol on the thyroid gland. In particular, subclinical hypothyroidism can occur after HSG with Lipiodol; and therefore, it is suggested to know the thyroid function before HSG with Lipiodol and to monitor the function with TSH and FT_4_ up to 6 months after HSG [23, 38–41]. Water-based CM have no, or a much smaller, effect on abdominal tissues and the thyroid gland.

CMSC guidelines are summarized in Table 3.

**Table 3:** Contrast agents and HSG: updated ESUR CMSC guidelines

	Level of evidence*
Approximately 10% more pregnancies and live births occur after HSG with oil-based CM compared to HSG with water-based CM	2
Image quality of HSG is significantly better when using oil-based CM	2
Intravasation during HSG occurs in equal amounts in both CM types	2
Oil-based CM can remain in the abdominal cavity for a prolonged period of time and show a significantly greater inflammatory effect on the peritoneum than water-based CM. The clinical consequences are unknown. Caution should be taken by using oil-based CM in HSG	3
Subclinical hypothyroidism occurs more often after HSG with oil-based CM. Therefore, in every woman receiving oil-based CM, thyroid function should be monitored before—6 months after HSG	3
Routine neonatal thyroid function tests after HSG are not indicated	3

*Level 1*: Systematic review of randomized trials or *n*-of-1 trials. *Level 2*: Randomized trial or observational study with dramatic effect including crossover studies. *Level 3*: Non-randomized controlled cohort/follow-up study. *Level 4*: Case-series, case-control, or historically controlled studies. *Level 5*: Mechanism-based reasoning. ^*^Grading of the literature was performed based on the Oxford Centre for Evidence Based Medicine (OCEBM) 2011 classification

## Supplementary Information

Below is the link to the electronic supplementary material.

Supplementary file1 (MP4 6525 KB)

Supplementary file2 (MP4 2161 KB)

## Abbreviations

CM: Contrast medium/media
CMSC: Contrast Media Safety Committee
ESUR: European Society of Urogenital Radiology
HSG: Hysterosalpingography
UF: Ultra Fluid

## Declarations

### Guarantor

The scientific guarantor of this publication is Remy Geenen.

### Statistics and biometry

No complex statistical methods were necessary for this paper.

### Study subjects or cohorts overlap

All study subjects or cohorts have been previously reported.

### Methodology

• retrospective literature analysis
